# Species identification, antibiotic resistance, and virulence in *Enterobacter cloacae* complex clinical isolates from South Korea

**DOI:** 10.3389/fmicb.2023.1122691

**Published:** 2023-03-23

**Authors:** Michidmaral Ganbold, Jungyu Seo, Yu Mi Wi, Ki Tae Kwon, Kwan Soo Ko

**Affiliations:** ^1^Department of Microbiology, Sungkyunkwan University School of Medicine, Suwon, Republic of Korea; ^2^Division of Infectious Diseases, Samsung Changwon Hospital, Sungkyunkwan University School of Medicine, Changwon, Republic of Korea; ^3^Department of Internal Medicine, School of Medicine, Kyungpook National University, Daegu, Republic of Korea

**Keywords:** *Enterobacter cloacae* complex, *Enterobacter hormaechei*, virulence, antibiotic resistance, species identification

## Abstract

This study aimed to identify the species of *Enterobacter cloacae* complex (ECC) isolates and compare the genotype, antibiotic resistance, and virulence among them. A total of 183 ECC isolates were collected from patients in eight hospitals in South Korea. Based on partial sequences of *hsp60* and phylogenetic analysis, all ECC isolates were identified as nine species and six subspecies. *Enterobacter hormaechei* was the predominant species (47.0%), followed by *Enterobacter kobei*, *Enterobacter asburiae*, *Enterobacter ludiwigii*, and *Enterobacter roggenkampii*. Multilocus sequence typing analysis revealed that dissemination was not limited to a few clones, but *E. hormaechei* subsp. *xiangfangensis*, *E. hormaechei* subsp. *steigerwaltii*, and *E. ludwigii* formed large clonal complexes. Antibiotic resistance rates were different between the ECC species. In particular, *E. asburiae*, *E. kobei*, *E. roggenkampii*, and *E. cloacae* isolates were highly resistant to colistin, whereas most *E. hormaechei* and *E. ludwigii* isolates were susceptible to colistin. Virulence was evaluated through serum bactericidal assay and the *Galleria mellonella* larvae infection model. Consistency in the results between the serum resistance and the *G. mellonella* larvae infection assay was observed. Serum bactericidal assay showed that *E. hormaechei*, *E. kobei*, and *E. ludwigii* were significantly more virulent than *E. asburiae* and *E. roggenkampii*. In this study, we identified the predominant ECC species in South Korea and observed the differences in antibiotic resistance and virulence between the species. Our findings suggest that correct species identification, as well as continuous monitoring is crucial in clinical settings.

## Introduction

*Enterobacter species are gram-negative*, *aerobic*, and *motile bacteria that belongs to the Enterobacteriaceae family. Enterobacter* spp. are ubiquitous and can be isolated from natural environments, animal hosts, and clinical environments (Sanders and Sanders, 1997; Davin-Regli et al., 2019). *Enterobacter* spp. is a member of the ESKAPE group, which is of particular concern, resulting in worse patient outcomes and increased treatment costs (Boucher et al., 2009). Many nosocomial and community-acquired infections are caused by *Enterobacter* spp., including urinary tract infections, respiratory infections, soft-tissue infections, osteomyelitis, and endocarditis, among others (Davin-Regli et al., 2019).

To date, 22 species have been identified in the genus *Enterobacter* (Parte et al., 2020; https://lpsn.dsmz.de/genus/enterobacter), but not all species are known to cause human disease (Davin-Regli et al., 2019). Seven species have been grouped into the *Enterobacter cloacae* complex (ECC); *Enterobacter asburiae*, *Enterobacter cloacae*, *Enterobacter hormaechei*, *Enterobacter kobei*, *Enterobacter ludwigii*, *Enterobacter mori*, and *Enterobacter nimipressuralis*. In addition, recently identified species, including *Enterobacter roggenkampii*, *Enterobacter chengduensis*, and *Enterobacter bugandensis*, are clustered with the species of ECC (Doijad et al., 2016; Sutton et al., 2018; Wu et al., 2019). Among them, *E. cloacae* and *E. hormaechei* are the most frequently isolated species in clinical infections, especially in immunocompromised patients and those hospitalized in intensive care units (Davin-Regli et al., 2019).

Because the ECC includes several species with varying antibiotic resistance and virulence, it is critical to identify the species. Though phenotype-based identification methods have been commonly used in clinical microbiology laboratories, they often fail to differentiate the species within the ECC. Although 16S rRNA gene sequencing is widely used for bacterial species identification, it has a limitation of poor discrimination ability among the ECC species (Hoffmann and Roggenkamp, 2003). Thus, many papers have mistakenly labeled the clinical isolates as *E. cloacae* or reported different species as ECC indiscriminately (Hong et al., 2018; Sutton et al., 2018). Meanwhile, *hsp60* gene sequences contain enough variation among the species of ECC for these distinctions to be made, indeed this gene has been recently utilized for ECC species identification (Hoffmann and Roggenkamp, 2003; Liu et al., 2022; Sato et al., 2022). However, there are few data on the species distribution of ECC and species characteristics (Liu et al., 2022), particularly in South Korea.

In this study, we identified species within the ECC of clinical isolates from eight hospitals in South Korea, based on *hsp60* gene analysis. We compared the antibiotic resistance and virulence features such as serum resistance and larvae infection among the species.

## Materials and methods

### Bacterial isolates

A total of 183 ECC clinical isolates were included in this study. They were collected between 2012 and 2021 from patients in eight hospitals in South Korea: 108 isolates (59.0%) from Samsung Medical Center (Seoul), 32 isolates (17.5%) from Samsung Changwon Hospital (Changwon), 18 isolates (9.8%) from Daegu Fatima Hospital (Daegu), 7 isolates (3.8%) from Chungnam National University Hospital (Daejeon), 6 isolates (3.3%) from Keimyung University Hospital (Daegu), 5 isolates (2.7%) from Changwon Fatima Hospital (Changwon), 4 isolates (2.2%) from Kyunghee University Hospital (Seoul), and 3 isolates (1.6%) from Chonnam National University Hospital (Gwangju). Among them, 161 isolates (88.0%) were from blood, followed by isolates from urine (13 isolates, 7.1%). Others were from sputum (3 isolates, 1.6%), wound (2 isolates, 1.1%), rectal swab (2 isolates, 1.1%), and tissue (1 isolate, 0.5%). The source of one isolate (0.5%) was unknown.

### Species identification

Species identification was performed by *hsp60* gene analysis as previously described (Hoffmann and Roggenkamp, 2003). The gene fragments were amplified and sequenced using primer set Hsp60-F/Hsp60-R. The obtained sequences of 264 bp were compared with the sequences of reference strains of 13 species or subspecies within the ECC, which were retrieved from the GenBank database (Supplementary Table 1). *hsp60* sequences were aligned with the ClustalW multisequence alignment program (Thompson et al., 1994). Phylogenetic trees were constructed using the neighbor-joining method and the MEGA 11.0 program package (Tamura et al., 2021) and iTOL software.

### Genotyping

For all ECC isolates, genotypes were determined using the Oxford multilocus sequence typing (MLST) scheme (Miyoshi-Akiyama et al., 2013). Seven housekeeping genes (*dnaA*, *fusA*, *gyrB*, *leuS*, *pyrG*, *rplB*, and *rpoB*) were amplified using primer sets according to the method previously reported. New alleles and new allelic profiles were submitted to the *E. cloacae* typing database^1^ and were assigned new numerical identifiers. Based on the determined allelic profiles of sequence types (STs), a minimum spanning (MS) tree was constructed using phyloviz v2.0a (Francisco et al., 2012) for all ECC isolates.

### Antibiotic susceptibility testing

For antibiotic susceptibility testing, 11 antibiotics were included: *ceftazidime* (CAZ), *cefepime* (CPM), *gentamicin* (GEN), *aztreonam* (AZT), *imipenem* (IMP), *meropenem* (MRP), *ciprofloxacin* (CIP), *colistin* (CL), *tigecycline* (TIG), *trimethoprim/sulfamethoxazole* (SXT), and *piperacillin/tazobactam* (P/T). Minimum inhibitory concentrations (MICs) were determined using the broth microdilution method, and susceptibility breakpoints were interpreted in accordance with the Clinical and Laboratory Standards Institute (CLSI) guideline (CLSI, 2021), except for tigecycline. For tigecycline, FDA-identified interpretive criteria for Enterobacteriaceae were used: susceptible (MIC, ≤2 mg/L), intermediate (MIC, 4 mg/L), and resistant (MIC, ≥8 mg/L; Pillar et al., 2008). *Escherichia coli* ATCC 25922 and *Pseudomonas aeruginosa* ATCC 27853 were used as control strains.

For carbapenem-resistant isolates, metallo-β-lactamase (MBL) and *Klebsiella pneumoniae* carbapenemase (KPC) genes were screened by PCR and sequencing (Lee et al., 2017).

### Serum bactericidal assay

Bacterial susceptibility to the bactericidal activity of serum was measured by evaluating the surviving bacterial cells after incubation in diluted serum, as described previously (Lee et al., 2018). Bacterial cultures were incubated until mid-log phase (OD_600_ of 0.5) in Luria-Bertani (LB) broth. One hundred microliters of culture were washed with 1× phosphate buffered saline (PBS) once and adjusted to a concentration of approximately 2 × 10^6^ bacteria per mL of 1 × PBS. Twenty-five microliters of the bacterial suspension were mixed with 75 microliters of pooled normal human serum (NHS; Innovative Research, MI, United States) and incubated, with shaking, for 3 h. After incubation, the mixture was washed once with 1 × PBS and diluted to spot onto an LB agar plate. The number of colony-forming units (CFUs) that survived after treatment with NHS was compared with the number of CFUs that survived after treatment with heat-inactivated serum for 30 min at 56°C.

### *Galleria mellonella* larval infection

For five *Enterobacter* species (*E. asburiae*, *E. kobei*, *E. ludwigii*, *E. hormaechei* subsp. *xiangfangensis*, and *E. roggenkampii*), *in vivo* virulence was investigated *via* infection of wax moth *G. mellonella* larvae (Tsai et al., 2011). Three isolates from each of the five species were selected based on the survival rate in the serum resistance assay (high, medium, and low). For the experiment, only healthy-looking larvae without melanization were used. *G. mellonella* larvae infection was performed by the intra-hemocoelic injection method through the last left pro-leg. Overnight-cultured bacteria were standardized to McFarland 0.5 with 10 mM phosphate-buffered saline (PBS, pH 6.5). Per group, 15 larvae were injected with 10 μL of bacterial suspension using a Hamilton syringe with a 30-gage, 8-mm needle. As control, equal number of larvae were injected with 10 μL of PBS in parallel to ensure that larval death was not due to injection trauma in each experiment. After injection, the larvae were placed in a petri dish and incubated at 37°C in the dark without food for 5 days (120 h). Every 24 h, the number of live larvae was evaluated; dead larvae were identified by the lack of motility and response to touch. Three independent experiments were performed for each isolate.

### Statistical analysis

Statistical analysis was performed using Prism version 8.00 for Windows (GraphPad Software, San Diego, CA). The differences in serum resistance were assessed using the student’s *t*-test, as well as the one-way ANOVA with Tukey multiple comparisons test. *Galleria mellonella* larvae survival was examined using the Kaplan–Meier method, and differences were determined by using the log-rank (Mantel Cox) test. *p*-values of less than 0.05 were considered statistically significant (^*^p < 0.05; ^**^p < 0.01; ^***^p < 0.001; ^****^p < 0.0001).

## Results

### Species identification

**Figure 1:**
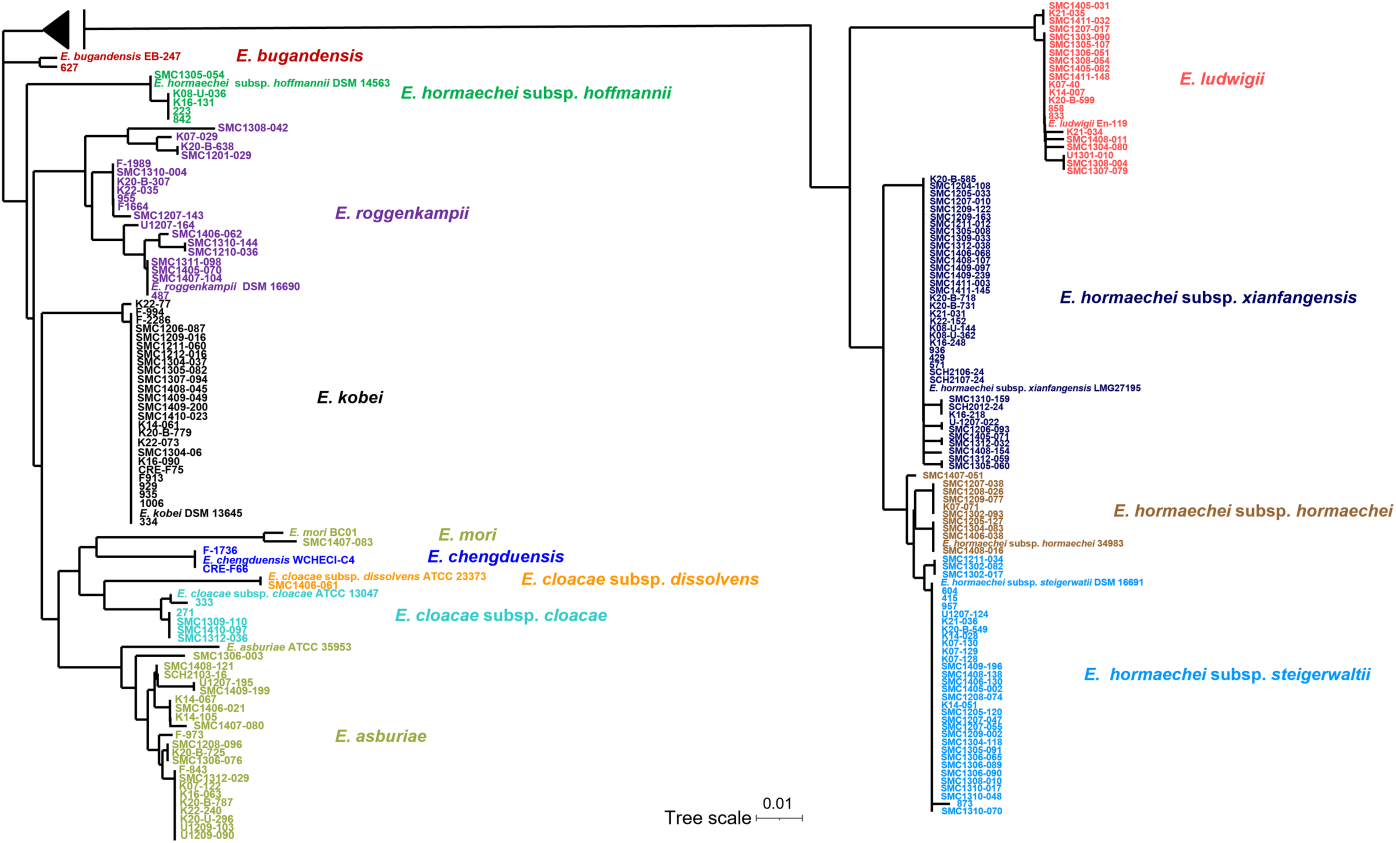
Phylogenetic tree based on neighbor-joining method using *hsp60* gene sequences of 183 clinical isolates and reference strains of *Enterobacter cloacae* complex (ECC). The sequences of the isolate and the reference strains were aligned using ClustalW option of mega 7.0 software program. A midpoint rooting option was applied to root the tree due to the absence of a reliable outgroup. The branch lengths are proportional to the changes in the nucleotides. Scale par indicates one substitution per 100 nucleotides.

Partial sequences of *hsp60* were used to identify the species of 183 ECC isolates collected from Korean hospitals. Species or subspecies could be identified clearly based on the *hsp60* sequence similarity with reference strains and phylogenetic grouping. As shown in Table 1 and Figure 1, *E. hormaechei* was identified the most frequently (86 isolates, 47.0%), followed by *E. kobei* (25 isolates, 13.7%), *E. asburiae* (23 isolates, 12.6%), *E. ludwigii* (21 isolates, 11.5%), and *E. roggenkampii* (18 isolates, 9.8%). *Enterobacter cloacae* and *E. chengduensis* were identified in six and two isolates, respectively, and one isolate each belonging to *E. bugandensis* and *E. mori*.

**Table 1:** Results of species identification based on partial sequences of *hsp60* gene for *Enterobacter cloacae* complex (ECC) isolates.

Species	*hsp60* Cluster^a^	No. of isolates (%)
*Enterobacter hormaechei*		86 (47.0)
subsp*. xianfangensis*	VI	38 (20.8)
subsp*. steigerwaltii*	VIII	33 (18.0)
subsp*. hormaechei*	VII	10 (5.5)
subsp*. hoffmannii*	III	5 (2.7)
*Enterobacter kobei*	II	25 (13.7)
*Enterobacter asburiae*	I	23 (12.6)
*Enterobacter ludwigii*	V	21 (11.5)
*Enterobacter roggenkampii*	IV	18 (9.8)
*Enterobacter cloacae*		6 (3.3)
subsp*. cloacae*	XI	5 (2.7)
subsp*. dissolvens*	XII	1 (0.5)
*Enterobacter chengduensis*	-^b^	2 (1.1)
*Enterobacter bugandensis*	IX	1 (0.5)
*Enterobacter mori*	-^b^	1 (0.5)
Total		183 (100)

a Cluster based on the grouping of Hoffmann and Roggenkamp (2003).

b *E. chengduensis* and *E. mori* have not been classified in Hoffmann and Roggenkamp (2003).

The *E. hormaechei* were classified into four subspecies: subsp. *xiangfangensis* (38 isolates, 20.8%), subsp. *steigerwaltii* (33 isolates, 18.0%), subsp. *hormaechei* (10 isolates, 11.6%), and subsp. *hoffmannii* (5 isolates, 2.7%; Table 1; Supplementary Table 2). A phylogenetic tree based on *hsp60* sequence similarity showed that *E. hormaechei* subsp. *hoffmannii* is not clustered with the other subspecies of *E. hormaechei* (Figure 1). Instead, *E. ludwigii* was closer to *E. hormaechei*. Among the six *E. cloacae* isolates, five isolates were classified as subsp. *cloacae* and the remaining one was subsp. *dissolvens*.

### Multilocus sequence typing analysis

**Figure 2:**
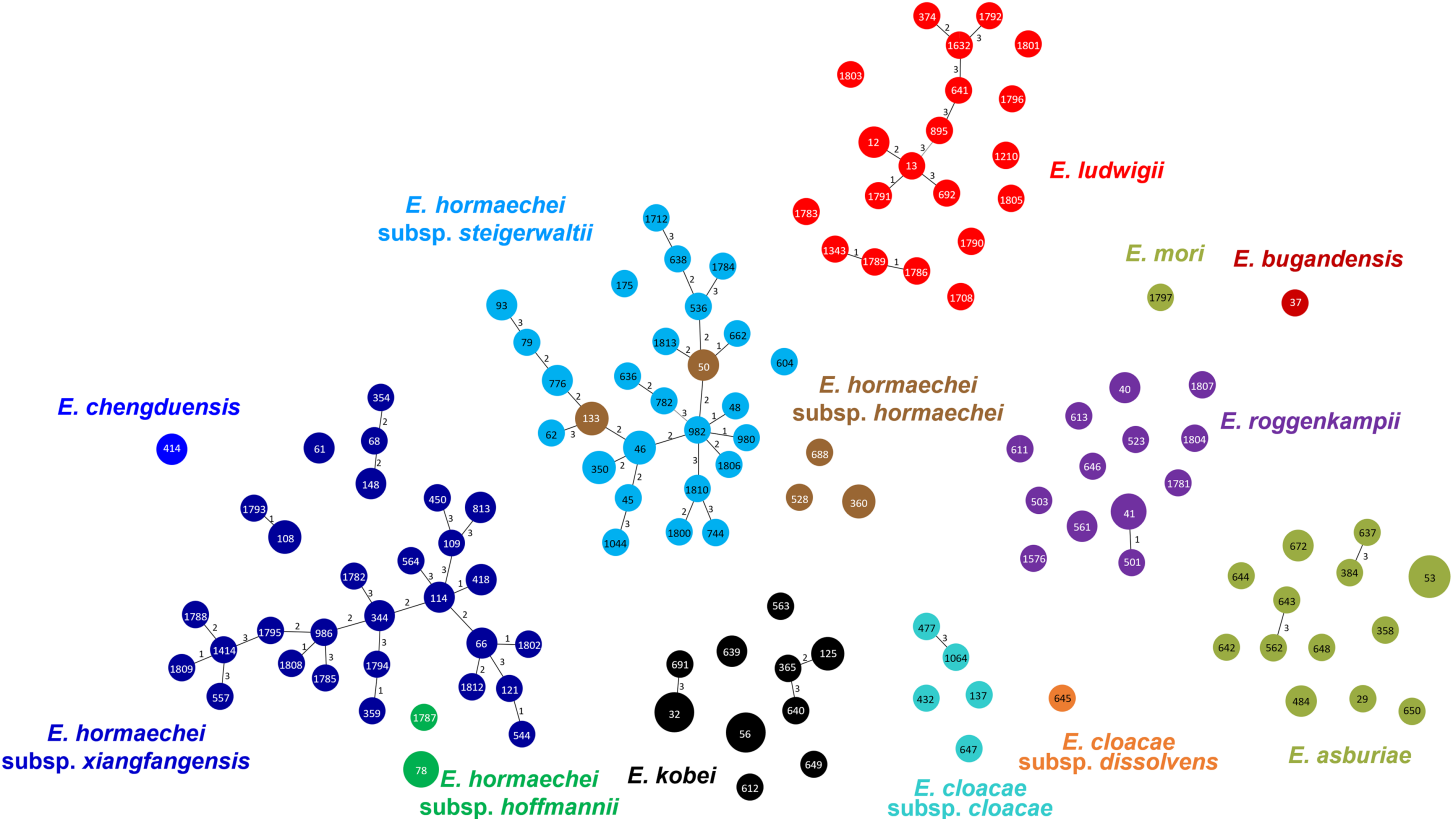
Minimum spanning tree of 183 clinical isolates of *Enterobacter cloacae* complex (ECC), based on the allelic profiles of multilocus sequence typing (MLST). Species based on the partial *hsp60* sequences are shown in different colors. Each node within the tree represents a single sequence type (ST), and the length of branches between each node represents the number of different alleles.

In MLST analysis, a total of 126 STs were identified among the 183 ECC isolates (Figure 2). Based on eBURST analysis, we designated STs sharing more than four alleles as clonal complexes. MS tree showed that large clonal complexes were formed in *E. hormaechei* subsp. *xiangfangensis*, *E. hormaechei* subsp. *steigerwaltii*, and *E. ludwigii*. However, *E. roggenkampii*, *E. kobei*, and *E. asburiae* isolates did not form large clonal complexes (Figure 2). Unexpectedly, MLST analysis revealed that some isolates of *E. hormaechei* subsp. *hormaechei*, which were designated as ST133 and ST50, were grouped with those of *E. hormaechei* subsp. *steigerwaltii*.

### Antibiotic resistance

For 183 ECC isolates, the trimethoprim/sulfamethoxazole resistance rate was the highest (62.8%), and colistin, ceftazidime, and aztreonam resistance rates were relatively high: 39.9, 39.3, and 32.8%, respectively (Table 2). In contrast, carbapenem (imipenem and meropenem) resistance rates were low, and tigecycline resistance was found in only one isolate.

**Table 2:** Antibiotic resistance in each species or subspecies of *Enterobacter cloacae* complex (ECC).

Species or subspecies	Antibiotic resistance [no. of isolates (%)]^a^										
	CAZ	CPM	GEN	AZT	IMP	MRP	CIP	CL	TIG	SXT	P/T
*Enterobacter hormaechei* (*n* = 86)	34 (39.5)	17 (19.8)	13 (15.1)	27 (31.4)	4 (4.7)	4 (4.7)	23 (26.7)	3 (3.5)	1 (1.1)	61 (70.9)	24 (27.9)
subsp*. xiangfangensis* (*n* = 38)	18 (47.4)	9 (23.7)	6 (15.8)	12 (31.6)	4 (10.5)	4 (10.5)	12 (31.6)	1 (2.6)	1 (2.6)	30 (78.9)	12 (31.6)
subsp*. steigerwaltii* (*n* = 33)	10 (30.3)	3 (9.1)	3 (9.1)	11 (33.3)	0	0	6 (18.2)	2 (6.1)	0	22 (66.7)	8 (24.2)
subsp*. hormaechei* (*n* = 10)	4 (40.0)	3 (30.0)	3 (30.0)	2 (20.0)	0	0	2 (20.0)	0	0	7 (70.0)	2 (20.0)
subsp*. hoffmannii* (*n* = 5)	2 (40.0)	2 (40.0)	1 (20.0)	2 (40.0)	0	0	3 (60.0)	0	0	2 (40.0)	2 (40.0)
*Enterobacter kobei* (*n* = 25)	8 (32.0)	3 (12.0)	2 (8.0)	6 (24.0)	1 (4.0)	1 (4.0)	5 (20.0)	24 (96.0)	0	12 (48.0)	4 (16.0)
*Enterobacter asburiae* (*n* = 22)	15 (65.2)	4 (17.4)	3 (13)	14 (60.9)	1 (4.3)	1 (4.3)	9 (39.1)	20 (86.9)	0	14 (60.9)	15 (65.2)
*Enterobacter ludwigii* (*n* = 21)	5 (23.8)	0	1 (4.8)	4 (19)	0	0	3 (14.3)	2 (11.1)	0	14 (66.7)	2 (9.5)
*Enterobacter roggenkampii* (*n* = 18)	6 (3.3)	0	0	5 (27.8)	0	0	5 (27.8)	15 (83.3)	0	10 (55.5)	5 (27.8)
*E. cloacae* (*n* = 6)	3 (50.0)	0	0	3 (50.0)	0	0	1 (16.7)	6 (100)	0	2 (33.3)	3 (50.0)
subsp*. cloacae* (*n* = 5)	3 (60.0)	0	0	3 (60.0)	0	0	1 (20.0)	5 (100)	0	2 (40.0)	3 (60.0)
subsp*. dissolvens* (*n* = 1)	0	0	0	0	0	0	0	1 (100)	0	0	0
*Enterobacter chengduensis* (*n* = 2)	1 (50.0)	2 (100)	2 (100)	1 (50.0)	1 (50.0)	1 (50.0)	2 (100)	2 (100)	0	1 (50.0)	1 (50.0)
*Enterobacter bugandensis* (*n* = 1)	0	0	0	0	0	0	0	1 (100)	0	0	0
*Enterobacter mori* (*n* = 1)	0	0	1 (100)	0	0	0	1 (100)	0	0	1 (100)	0
Total (*n* = 183)	72 (39.3)	26 (14.2)	22 (12.0)	60 (32.8)	7 (3.8)	7 (3.8)	49 (26.8)	73 (39.9)	1 (0.5)	115 (62.8)	54 (29.5)

CAZ, *ceftazidime*; CPM, *cefepime*; GEN, *gentamicin*; AZT, *aztreonam*; IMP, *imipenem*; MRP, *meropenem*; CIP, *ciprofloxacin*; CL, *colistin*; TIG, *tigecycline*; SXT, *trimethoprim/sulfamethoxazole*; P/T, *piperacillin/tazobactam*.

Some antibiotic resistance rates were different between the species of ECC. While colistin resistance rates were high in *E. cloacae* (100%), *E. kobei* (96.0%), *E. asburiae* (86.9%), and *E. roggenkampii* (83.3%), they were low in *E. hormaechei* (3.5%) and *E. ludwigii* (11.1%; Table 2; Supplementary Figure 1). The ceftazidime resistance rate was lower in *E. roggenkampii* (3.3%) than in the other ECC species. *E. ludwigii* isolates showed a low piperacillin/tazobactam resistance rate (9.5%) compared to that of the other species, including *E. asburiae* (65.2%) and *E. cloacae* (50.0%).

Subspecies of *E. hormaechei* showed different resistance rates to some antibiotics. *E. hormaechei* subsp. *steigerwaltii* showed low resistance to cefepime and gentamicin (Table 2; Supplementary Figure 1). The ciprofloxacin resistance rate was higher in *E. hormaechei* subsp. *hormaechei* than in that of the other subspecies. Isolates resistant to imipenem and meropenem were found only in *E. hormaechei* subsp. *xiangfangensis*, in addition to *E. chengduensis*.

Among the seven carbapenem-resistant ECC isolates, MBL or KPC genes were identified in six isolates. *bla*_NDM-1_ and *bla*_NDM-5_ were found in one and two of the *E. hormaechei* subspecies *xiangfangensis* isolates, respectively. *bla*_IMP-1_ was detected in one *E. hormaechei* subsp. *xiangfangensis* isolate. *bla*_KPC-2_ was identified in an isolate of *E. asburiae* and both *bla*_IMP-1_ and *bla*_VIM-2_ were found in an isolate of *E. kobei*. No MBL or KPC genes were identified in a carbapenem-resistant *E. chengduensis* isolate.

Among the 183 colistin-resistant ECC isolates, *mcr-10* was detected in all, and *mcr-9* was identified in only 11 isolates (6.0%).

### Bacterial survival against normal human serum

**Figure 3:**
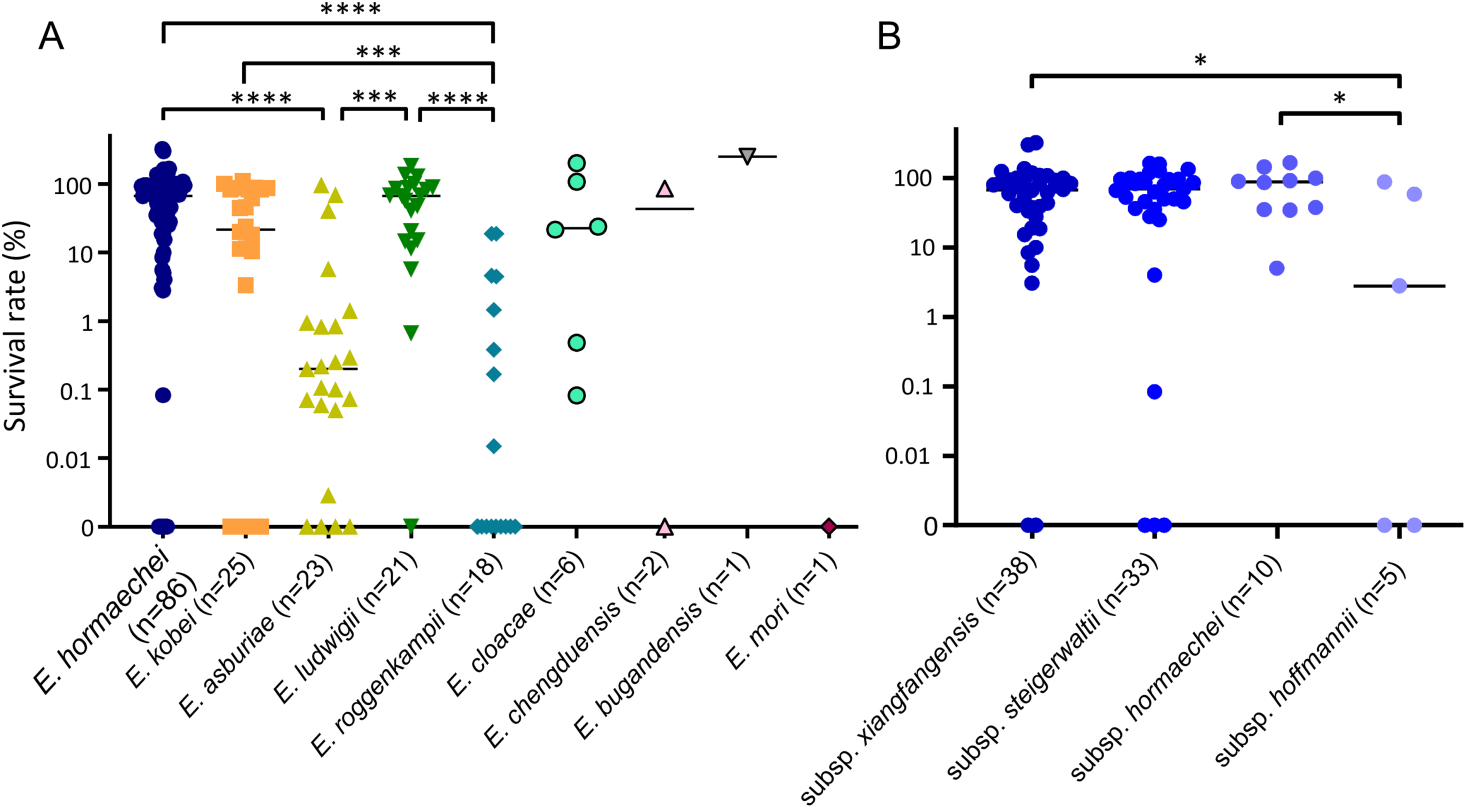
Results of serum bactericial assay among *Enterobacter cloacae* complex (ECC) species **(A)** and subspecies of *Enterobacter hormaechei* **(B)**. Bacterial survival rates were determined after 3 h of incubation with normal human serum (NHS). Heat-inactivated serum (HIS) was used as a negative control. ^*^p < 0.05; ^***^p < 0.001; ^****^p < 0.0001.

For all ECC isolates, we measured the survival rates of bacterial isolates against NHS and compared them by species (Figure 3A). Although the survival rates against serum were diverse in ECC isolates (0–135.7%), they also differed significantly among the species or subspecies. The serum resistance levels of *E. roggenkampii* isolates were significantly lower than in *E. hormaechei*, *E. kobei*, or *E. ludwigii* isolates (Figure 3A). In addition, *E. asburiae* isolates also showed lower survival rates against serum than *E. hormaechei* or *E. ludwigii* isolates. That is, *E. hormaechei*, *E. kobei*, and *E. ludwigii* isolates generally exhibited higher serum resistance rates than *E. asburiae* or *E. roggenkampii* isolates, despite considerable variations. In particular, 10 out of 18 *E. roggenkampii* isolates (55.6%) were completely killed by human serum within 3 h.

We also compared serum resistance among the subspecies of *E. hormaechei* (Figure 3B). The subsp. *xianfangensis* and subsp. *hormaechei* isolates showed significantly higher serum resistance rates than subsp. *hoffmannii* isolates.

### Survival of *Galleria mellonella* larvae against bacterial isolates

We evaluated the survival of *G. mellonella* larvae against five ECC species: *E. hormaechei* subsp. *xianfangensis*, *E. kobei*, *E. asburiae*, *E. ludwigii*, and *E. roggenkampii*. For each species, three isolates were selected based on the results of the serum resistance assay: high (near 100% or above), intermediate (0.1–90%), and low (0%). For *E. roggenkampii*, only two isolates were included in this experiment, because no isolates showed a high survival rate in the serum bactericidal assay.

**Figure 4:**
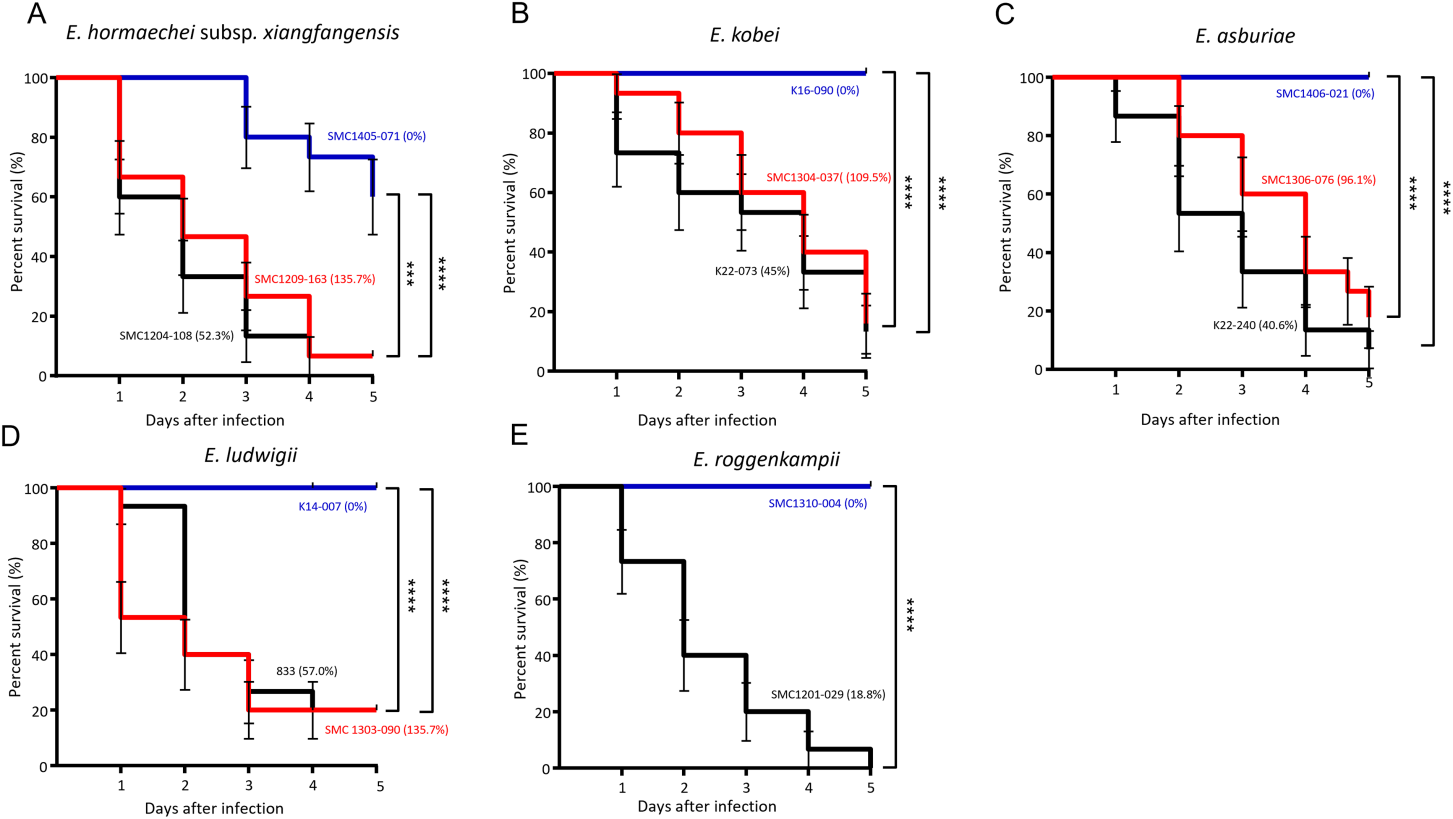
Results of *Galleria mellonella* larvae infection experiments. Survival of larvae infected with three isolates from each of the five *Enterobacter cloacae* complex (ECC) species, except *Enterobacter roggenkampii*. *Enterobacter hormaechei* subsp. *xianfangensis* **(A)**, *Enterobacter kobei* **(B)**, *Enterobacter asburiae* **(C)**, *Enterobacter ludwigii* **(D)**, and *Enterobacter roggenkampii* species **(E)**. Three isolates from each species were selected for low (blue line), intermediate (black line), and high (red line) survival rates in the serum bactericidal assay. For *E. roggenkampii*, with no isolates showing high survival rate in serum bactericidal assay, two isolates were included. The percentages in parentheses represent the survival rates in the serum bactericidal assay. Five larvae were evaluated per isolate, and results were obtained from three independent experiments. *p* < 0.001; ^****^p < 0.0001.

In all five ECC species, *G. mellonella* larvae showed higher survival rates against isolates with significantly high serum resistance rates (Figure 4). Except for *E. hormaechei*, four isolates that were completely eradicated by the serum did not kill any *G. mellonella* larvae. However, no difference in survival rates of *G. mellonella* larvae was found between the isolates with intermediate and high serum resistance rates. Regardless of bacterial species, throughout the 5 days of infection, most larvae were killed by the isolates with intermediate and high resistance rates against serum within 5 days of infection.

## Discussion

ECC has been repeatedly reported as a nosocomial pathogen (Hoffmann and Roggenkamp, 2003; Kremer and Hoffmann, 2012; Beyrouthy et al., 2018; Davin-Regli et al., 2019). Although diverse ECC species has been involved in human infections, no routine phenotypic methods can differentiate ECC species from one another, because these tests are unreliable and irreproducible (Paauw et al., 2008). Instead, genotypic methods based on partial hsp60 sequences have been reported to be useful for identifying ECC species (Hoffmann and Roggenkamp, 2003). In this study, we identified ECC species and subspecies in clinical isolates from South Korea using partial *hsp60* sequences, and compared their virulence.

In a recent paper, Wu et al. suggested that *E. hormaechei* subsp. *hoffmannii* and *E. cloacae* subsp. *dissolvens* are distinct species rather than subspecies of *E. hormaechei* and *E. cloacae*, respectively, based on genome analysis (Wu et al., 2020). In addition, these researchers insisted that *E. hormaechei* is synonym of *E. xiangfangensis*, which is not a subspecies of *E. hormaechei*. We also found that isolates of *E. hormaechei* subsp. *hoffmannii* represented a distinct group and were not clustered with other subspecies of *E. hormaechei* and that isolates of the two subspecies of *E. cloacae* showed enough *hsp60* sequence dissimilarity to be represented as separate species. In addition, some isolates of *E. hormaechei* subsp. *hormaechei* were grouped into the cluster of *E. hormaechei* subsp. *steigerwaltii* in MLST analysis. However, the proposal by Wu et al. has not been approved officially in the List of Prokaryotic names withstanding in Nomenclature (LPSN, https://www.bacterio.net/; Parte et al., 2020). Thus, we used the official nomenclature of the genus *Enterobacter* in this study.

In the present study, diverse species of ECC were identified in patients, including four recently described species (*E. bugandensis*, *E. chengduensis*, *E. roggenkampii*, and *E. mori*; Zhu et al., 2011; Doijad et al., 2016; Sutton et al., 2018; Wu et al., 2019). Among the ECC isolates from South Korea, *E. hormaechei* was the predominant species, as was also the case in a recent study from China (Liu et al., 2022). Unlike the results from China, *E. hormaechei* subsp. *xiangfangensis* was the most predominant among the subspecies of *E. hormaechei*. None of the *E. hormaechei* subsp. *xiangfangensis* isolates were identified in the Chinese study (Liu et al., 2022). Since *E. hormaechei* was first identified in 1989 (Ohara et al., 1989), it has been reported from natural environments, and has recently emerged worldwide as ECC nosocomial pathogen (Yeh et al., 2022). Despite being the most frequently isolated species of *Enterobacter* in South Korea, as determined by our study, *E. hormaechei* has only occasionally been reported. Two explanations are possible: (i) *E. hormaechei* may have recently been introduced into South Korea and has disseminated, (ii) species identification within the genus *Enterobacter* has not been performed, and only *E. cloacae* or ECC was reported (Lee et al., 2017; Jeon et al., 2021; Kim et al., 2022). Regardless, as shown in this study, antibiotic resistance and virulence are different for each species within the ECC, thus, a definite identification of the species is required.

In all ECC species or subspecies, no extensive dissemination of a particular clone was identified. On the other hand, eBURST analysis showed that most isolates of *E. hormaechei* subsp. *hoffmannii* and *E. hormaechei* subsp. *steigerwaltii* clustered into large clonal complexes, but other subspecies did not. This may imply that the evolution or the spread of each species of ECC is different. The two subspecies of *E. hormaechei* forming large clonal complexes seem to have been differentiated into many genotypes for a considerable period since its introduction in South Korea. Thus, the “recent introduction” explanation of why *E. hormaechei* has not been previously reported in South Korea may not be plausible.

Regarding antibiotic resistance, the first to note is that most of the carbapenem-resistant isolates were identified as *E. hormaechei* subsp. *xiangfangensis*. A clone of *E. hormaechei* subsp. *xiangfangensis*, ST171, which has been recognized as one of the globally emerging carbapenemase-producing ECC clones (Pereira et al., 2019; Karlsson et al., 2022). Although the ST171 clone was not identified in this study, it is necessary to monitor the spread of MBL-producing *E. hormaechei* subsp. *xiangfangensis* isolates (Roberts et al., 2020; Yeh et al., 2022). It is also noteworthy that colistin resistance was identified infrequently among the four subspecies of *E. hormaechei* and *E. ludwigii*, whereas most isolates of *E. kobei*, *E. asburiae*, *E. roggenkampii*, and *E. cloacae* were resistant to colistin. Although further studies are needed, it is likely that colistin, which has been suggested as one of the last resorts to treat multidrug-resistant gram-negative pathogens, may not be effective against some species of ECC. Thus, it is necessary to carefully select antibiotics according to the species identification results for ECC isolates.

We evaluated the virulence in species or subspecies of ECC using two methods: bacterial survival against normal human serum and survival of *G. mellonella* larvae against bacteria. The serum bactericidal assay has been used for assessing the virulence of bacteria (Di Pilato et al., 2022), and *G. mellonella* larvae have been used for studying bacterial virulence due to their sophisticated cellular and humeral defenses, ease of maintenance, and exemption from ethical limitations (Yang et al., 2017). In this study, we identified the association between bacterial survival against serum and the survival of *G. mellonella* larvae against bacteria. That is, high survival of *G. mellonella* larvae—that is, low bacterial virulence—was shown against the ECC isolates with low bacterial survival against serum. In addition, our results on virulence indicated that it is not desirable to select and measure only some strains in order to determine the overall virulence of a specific species or group.

Based on the serum bactericidal assay on all of the ECC isolates included in this study, we found that *E. asburiae* and *E. roggenkampii* were less virulent than the other ECC species, *E. hormaechei*, *E. kobei*, and *E. ludwigii*. This result is inconsistent with a previous study that measured the number of virulence genes carried by isolates; in that work, *E. kobei* and *E. ludwigii* carried fewer virulence genes (Liu et al., 2022). This means that the virulence of each ECC species may not be related to the number of virulence genes. Not long ago, *E. cloacae* was considered the most clinically important species among the ECC, and species such as *E. asburiae* and *E. kobei* were reported to be rarely found in human patients (Mezzatesta et al., 2012). As shown in recent studies, including our results, diverse ECC species are associated with human infections, and their pathogenicity is being re-evaluated (Davin-Regli et al., 2019). In order to confirm the results of this study, it is necessary to perform a clinical study on the difference in patient severity or treatment results according to species of ECC.

Our study has some limitations. Although ECC isolates were collected from multiple hospitals, insufficient numbers and uneven distribution of the isolates may affect the overall assessment of species distribution, antibiotic resistance, and virulence.

In the present study, we identified species of ECC isolates from South Korea based on partial *hsp60* sequences. *E. hormaechei* was predominant, followed by *E. kobei*, *E. asburiae*, *E. ludwigii*, and *E. roggenkampii*. The *E. hormaechei* isolates were differentiated into several subspecies. The species or subspecies of ECC represented different antibiotic resistance and virulence. In particular, high colistin resistance rates were associated with *E. kobei*, *E. asburiae*, *E. roggenkampii*, and *E. cloacae*. Low virulence with respect to serum susceptibility was observed in *E. asburiae* and *E. roggenkampii*. This suggests that definite species identification and continuous monitoring should be required in clinical settings.

## Data availability statement

The datasets presented in this study can be found in online repositories. The names of the repository/repositories and accession number(s) can be found in the article/Supplementary material.

## Author contributions

MG performed the experiments, analyzed the data, and wrote the manuscript. JS performed the experiments and analyzed the data. YW and KiK provided the experimental materials and analyzed the data. KwK designed the experiments, analyzed the data, and wrote the manuscript. All authors contributed to the article and approved the submitted version.

## Funding

This research was supported by the Basic Science Research Program through the National Research Foundation of Korea (NRF), funded by the Ministry of Science and ICT (grant number NRF-2022R1A2B5B02001716).

## Conflict of interest

The authors declare that the research was conducted in the absence of any commercial or financial relationships that could be construed as a potential conflict of interest.

## Publisher’s note

All claims expressed in this article are solely those of the authors and do not necessarily represent those of their affiliated organizations, or those of the publisher, the editors and the reviewers. Any product that may be evaluated in this article, or claim that may be made by its manufacturer, is not guaranteed or endorsed by the publisher.

## Footnotes

^1^ https://pubmlst.org/organisms/enterobacter-cloacae
