# Supplementary material for: Species identification, antibiotic resistance, and virulence in Enterobacter cloacae complex clinical isolates from South Korea
Source: Front Microbiol. 2023 Mar 23;14:1122691. doi: 10.3389/fmicb.2023.1122691 (PMC10076837; doi:10.3389/fmicb.2023.1122691)
Supplement: Supplementary file 1 [file Table_1.pdf]

Supplementary Table S1. Strain and accession number of *hsp60* sequences used for species identification of the *Enterobacter cloacae* complex (ECC) isolates

Species or subspecies	Strain Number	Accession no.
E. asburiae	ATCC 35953	CP011863
E. bugandensis	EB-247	NZ_LT992502.1
E. chengduensis	WCHECI-C4	NZ_CP043318
E. cloacae subsp. *cloacae*	ATCC 13047	NC_014121
E. cloacae subsp. *dissolvens*	ATCC 23373	NZ_WJWQ01000001
E. hormaechei subsp. *hoffmannii*	DSM 14563	CP017186
E. hormaechei subsp. *hormaechei*	34983	CP010377
E. hormaechei subsp. *steigerwaltii*	DSM 16691	CP017179
E. hormaechei subsp. *xiandfangensis*	DSM LMG 27195	CP017183
E. kobei	DSM 13645	CP017181
E. ludwigii	En-119	CP017279
E. mori	BC01	CP084692
E. roggenkampii	DSM 16690	CP017184

Supplementary Table S2. Primer sequences used in this study.

Primer	Sequences
hsp60-F	GGTAGAAGAAGGCGTGGTTGC
hsp60-R	ATGCATTCGGTGGTGATCATCAG
dnaA-FW	AACCCGCTGTTCCTCTATGGCGGC
dnaA-RV	GCCAGCGCCATCGCCATCTGACGCG
fusA-FW	AGTAGAACGTTCCATGCGTGTTCT
fusA-FW	TCTTTCAGACCGATAGCTGCAGC
gyrB-FW	TCGACGAAGCGCTCGCGGGTCACTG
gyrB-RV	GCAGAACCGCCCGCGGAGTCCCCT
leuS-FW	TCCTGCCGGAAGATGTGGTCATGGA
leuS-RV	ATAGCCGCAATTGCGGTATTGAAGGTC
pyrG-FW	TCGGTAGTCTGAGGTTCCTTATGT
pyrG-RV	TGTTATTTATCCCCTTGCCGCACG
rplB-FW	GTAAACCGACATCTCCGGGTC
rplB-RV	ACCTTTGGTCTGAACGCCCCACGGA
rpoB-FW	AAAAACGTATTCGTAAGGA
rpoB-RV	CTCAGCTTAGCCAGCAGATCCAGG
KPC-FW	GTATCGCCGTCTAGTTCTGC
KPC-RV	GGTCGTGTTTCCCTTTAGCC
IMP-FW	GAAGGYGTTTATGTTCATAC
IMP-RV	GTAMGTTTCAAGAGTGATGC
NDM-FW	CCAATATTATGCACCCGGTCG
NDM-RV	ATGCGGGCCGTATGAGTGATTG
VIM-FW	GTTTGGTCGCATATCGCAAC
VIM-RV	AATGCGCAGCACCAGGATAG
SIM-FW	TACAAGGGATTCGGCATCG
SIM-RV	TAATGGCCTGTTCCCATGTG
SPM-FW	CTGCTTGGATTCATGGGCGC
SPM-RV	CCTTTTCCGCGACCTTGATC
GIM-FW	TCGACACACCTTGGTCTGAA
GIM-RV	AACTTCCAACTTTGCCATGC

Supplementary Table S3. The similarity of partial *hsp60* sequences between reference strains and clinical isolates of subspecies of *E. hormaechei*

Subspecies	subsp. *xiangfangensis* LMG27195	subsp. *steigerwaltii* DSM 16691	subsp. *hormaechei* 34983	subsp. *hoffmannii* DSM 14653
subsp. *xiangfangensis*	**99.62–100 %**	97.72–98.10%	97.13–98.10%	94.32–94.69%
subsp. *steigerwaltii*	97.72–98.10%	**99.62%–100%**	98.86–99.24%	93.56–94.32%
subsp. *hormaechei*	97.72%	99.24%	**99.24–100%**	94.32–94.69%
subsp. *hoffmannii*	94.31–94.69%	94.32–94.67%	93.94–94.32%	**99.62–100%**
